# The Influence of Branching Degree and Temperature on the Relaxation of Semidilute and Concentrated Aqueous Solutions of Pectins Obtained from Red- and Blackcurrant

**DOI:** 10.3390/molecules31071121

**Published:** 2026-03-28

**Authors:** Michał Pancerz, Anna Ptaszek

**Affiliations:** 1 Department of Engineering and Machinery in Food Industry, Faculty of Food Technology, University of Agriculture in Krakow, Balicka 122, 30-149 Krakow, Poland; michal.pancerz@urk.edu.pl; 2 Centre for Innovation and Research on Prohealthy and Safe Food, University of Agriculture in Krakow, Balicka 104, 30-149 Krakow, Poland

**Keywords:** blackcurrant, redcurrant, pectin, relaxation, branching

## Abstract

Pectins are structurally complex plant polysaccharides whose functional properties strongly depend on molecular structure that may vary depending on the source of origin. The present study aimed to characterize and compare the hydrodynamic properties of pectins obtained from red and blackcurrants in semidilute and concentrated aqueous solutions. Pectins were extracted and analyzed using light scattering methods and rheology at 25 °C, 30 °C, 35 °C and 40 °C. The methodology used enabled the determination of the hydrodynamic properties of the pectins with changing temperature and concentration, and mathematical modeling was performed using the Kohlrausch–Williams–Watts model. The obtained samples differed in molecular structure, and these differences were reflected in the chain behavior in aqueous solution. The results indicate that even closely related botanical sources may yield pectins with significantly different functional properties. Hydrodynamic studies revealed that relaxation phenomena occurred in a similar manner for redcurrant pectin in the concentrated region and for blackcurrant pectin in the semidilute region (similar diffusion coefficients). Under shear flow conditions, blackcurrant pectin solutions behaved like Newtonian fluids, whereas redcurrant pectin exhibited complex, non-Newtonian behavior. Redcurrant pectin solutions also exhibited lower apparent viscosity values at concentrations comparable to those of blackcurrant pectin. The ability to scale apparent viscosity values indicated a unchanging friction mechanism in viscous flow, characteristic of semidilute and concentrated regions.

## 1. Introduction

**Figure 1:**
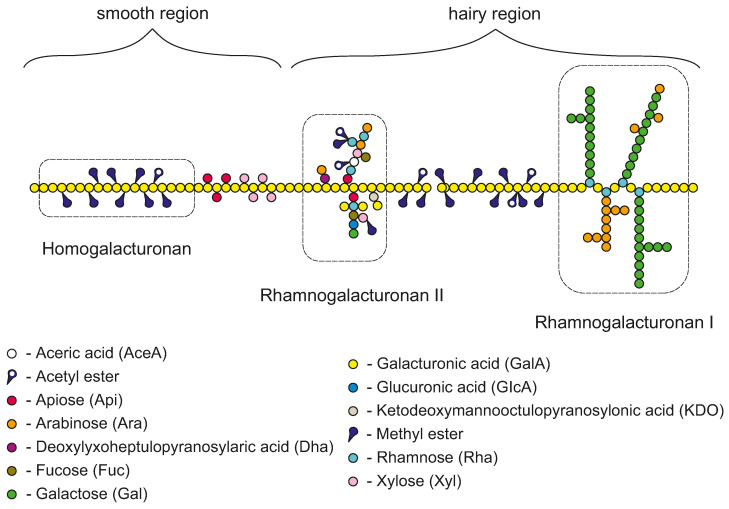
General structure of pectin chain, schematic diagram according to [2].

Pectins are heteropolysaccharides of a complex nature widely distributed in the primary cell walls and middle lamella of higher plants. Their structure consists of three structurally different segments: homogalacturonan (HG), rhamnogalacturonan I (RGI) and rhamnogalacturonan II (RGII), which together form a complex structure of this polysaccharide [1]. A simplified diagram of the chain structure is shown in Figure 1.

HG is a relatively linear chain built from α−(1→4)-linked D-galacturonic acid monomers often interrupted by the presence of short side chains composed of simple sugars. Some of the galacturonic acid residues of HG may be esterified with methanol. RGI and RGII consist of a repeating disaccharide backbone built of rhamnose and galacturonic acid units substituted to varying degrees with glycan side chains [3]. The presence of differentiated chain fragments in the pectin molecule results in two characteristic structural regions that can be distinguished (Figure 1): a linear *smooth* region, composed mainly of HG without side branches, and a highly branched *hairy* region consisting of RGI and RGII segments that are highly spatially expanded [4]. The functional properties and behavior of the pectin chain in aqueous solutions and consequently the possibilities for practical application in industry are determined by the molecular structure of the chain. In particular, the degree of esterification of the homogalacturonan chain and the ratio of *smooth* to *hairy* regions determine solubility, chain conformation, and hydrodynamic behavior in aqueous solutions. These parameters are crucial for technologically important properties such as viscosity, water binding and gel-forming ability, and therefore the possibility of use in specific food industry processes [5]. Pectin is a polyelectrolyte due to the presence of ionizable carboxyl groups in galacturonic acid residues. Consequently, the physicochemical properties of pectin solutions are strongly dependent on quality of the solvent used. Variations in ionic strength (salt addition) or a change in pH will affect the distribution of ionic charges that keeps the molecule in an extended form. This can alter electrostatic interactions along the polymer backbone, affecting chain conformation and intermolecular interactions, which in turn influence the rheological properties and viscosity of the solution [6]. The molecular structure can also be described by determining characteristic indicators, which are the molar ratios of sugars in the polysaccharide chain. The R1 ratio (galacturonic acid to neutral sugars involved in side chains) indicates the linearity of the pectin chain. R2, on the other hand, determines the proportion of rhamnose to galacturonic acid in the pectin chain $\left(\frac{Rha}{GalA}\right)$, reflecting the contribution of RG to the entire pectin population, thereby determining the number of side branches of the pectin chain that correspond to amount of *hairy regions* in the pectin chain. R3 $\left(\frac{Ara+Gal}{Rha}\right)$ comparesthe amount of side chains of RGI to rhamnose, reflecting the extent of branching of RGI and providing information about the size and volume of the side chains of the pectin molecule [7]. From a technological perspective, important for the food industry, pectin is defined as a polysaccharide containing more than 65% galacturonic acid [8]. It is most commonly obtained from citrus or apple pomace and standardized to obtain reproducible functional properties [3]. For this reason, two main types of pectins are distinguished in the literature: high-methoxyl (HM) and low-methoxyl (LM). This classification is based on the degree of substitution of galacturonic acid residues with methanol in the pectin chain. This is the classification most commonly used because the degree of esterification determines the gelation mechanism and conditions, which is the most important functional property in industrial applications [9]. However, the physicochemical properties of pectin, such as molecular weight, chain length, and the ratio of HG to RG domains (*smooth* and *hairy* regions of the chain), vary depending on the source. Numerous studies report that significant changes in the structure of pectin occur as fruits and vegetables ripen. Therefore, the choice of raw material and its maturity stage may influence its industrial applications by obtaining various hydrodynamic properties and a diverse chain structure [10,11,12]. Despite extensive research on commercial citrus and apple pectins, much less attention has been paid to other potential sources. Currants are nutritionally valuable fruits, but they are used almost exclusively to make juices and jams, generating substantial by-products that may serve as a promising alternative source of pectin [13]. Previous studies of selected properties of pectins obtained from blackcurrants and redcurrants revealed differences in their structure [14,15], which are reflected in their ability to form a gel [2]. However, no comparison has yet been made of the hydrodynamic properties of solutions in the semidilute and concentrated ranges, nor has any attempt been made to model relaxation phenomena or rheological properties in the flow regime.

The aim of this study was to characterize and compare the relaxation phenomena of pectins isolated from red and blackcurrants in aqueous solution. In particular, this study aimed to check whether pectins obtained from botanically related but distinct currant species differ in hydrodynamic and rheological behavior and whether differences in molecular structure may influence their potential industrial applicability. Attempts were made to apply the time–temperature rule to the apparent viscosity as a function of concentration regime and model the rheological properties mathematically.

## 2. Results

The molecular properties of red and blackcurrant pectins are presented in Table 1. For redcurrant pectin, DE = 57.1%, and for blackcurrant, DE = 67.4%. Both pectins can be considered highly methylated, so their behavior in solution will be shaped by the same mechanisms, and the differences will result from the structure of the chain. The molecular weight of pectin isolated from redcurrants is almost 10 times higher than that of pectin from blackcurrants and amounts to 1020 kg·mol^−1^. This is a high value compared to most commonly used apple and citrus pectins, whose masses, according to [16] are $M_{w}$ = 194 kg·mol^−1^ for citrus and $M_{w}$ = 268 kg·mol^−1^ for apple. As reported in the literature, such high-molecular-weight values are characteristic for water-soluble pectin fraction obtained via simple aqueous extraction [17]. The high molar mass of the redcurrant pectin chain is accompanied by strong linearity, as indicated by the high R1 = 2.27 (share of HG in pectin chain) and low R2 = 0.06 (RG contribution to the pectin population). Furthermore, the degree of branching of the few *“hairy”* regions of the redcurrant pectin chain was not significant (R3 is 1.03). This may mean that redcurrant pectin is characterized by a very long, mostly linear chain, whose few side chains are also almost linear. Blackcurrant pectin, on the other hand, is characterized by a low molecular weight ($M_{w}$ = 116 kg·mol^−1^), moderate HG content (R1 = 1.50), and a high proportion of RG in the chain (R2 = 0.21). In addition, the side chains are highly branched (R3 = 8.77). Molecular properties strongly influence the hydrodynamic properties of pectin in solutions. Short, branched chains with low molar weights will be much more easily hydrated than strongly coiled, long chains with few spatial hindrances, facilitating water penetration [6].

**Figure 2:**
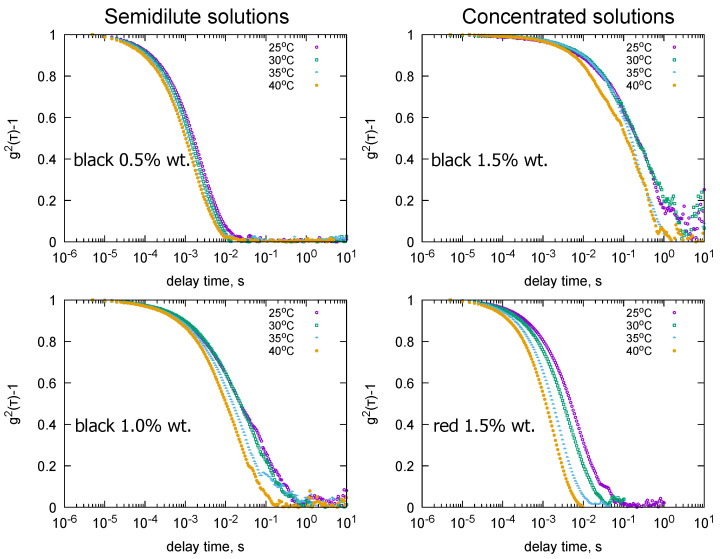
Experimental autocorrelation functions ($g^{2}\left(\tau \right)-1$) at different temperatures (25 °C, 30 °C, 35 °C, 40 °C) for pectin aqueous solutions of selected concentrations. Left column: blackcurrant pectin in the semidilute regime at 0.5% and 1.0%, right column: concentrated solutions at 1.5% of blackcurrant and redcurrant pectin. Each curve shows the temperature-dependent relaxation behavior of pectin in water.

The hydrodynamic properties of pectin solutions are presented in Figure 2. For blackcurrant pectin solutions in the semidilute concentration range, doubling the concentration resulted in a change in hydrodynamic behavior. For a 0.5% wt solution, the autocorrelation function disappeared in the range of 0.01 to 0.1 s, regardless of the measurement temperature. A twofold increase in concentration altered the relaxation pattern of the solution. Firstly, the temperature affected the course of the autocorrelation function, and the function disappeared in the range of 0.1 to 1 s. For a 1.5% solution (concentrated regime), the autocorrelation function at 25 °C and 30 °C showed a clear increase in the decay time of the autocorrelation function to 10 s. Different behavior was observed in the case of a 1.5% redcurrant pectin solution (concentrated regime). The behavior of the solution was clearly differentiated by temperature, but the decay times varied between 0.01 and 0.1 s (similar to those of the 0.5% blackcurrant pectin solution). Pectins obtained from blackcurrants had an average molecular weight of 116 kDa. Only in the case of the solution with the lowest concentration could the values of the KWW model be estimated (relaxation times and the proportion of fast relaxation processes, Equation (1)). This made it possible to determine the values of the fast and slow mode diffusion coefficients (Table 2). Based on these values, only the $R_{h}$ values for the fast mode were calculated. The values varied within the range of 150 to 490 nm. The proportion of fast relaxation processes changed from 69% at 25 °C to 28% at 40 °C as the measurement temperature increased. For the slow mode, relaxation times were determined, and diffusion coefficients were estimated on the basis of these. These ranged from 2.14 $\times \;10^{-13}\frac{{\mathrm{cm}}^{2}}{s}$ to 7.29 $\times \;10^{-13}\frac{{\mathrm{cm}}^{2}}{s}$, indicating that the hydrodynamic radii of the pectin chains responsible for the slow mode were greater than 500 nm (Table 2). Their proportion, which was less than 50% at each temperature, did not determine the relaxation phenomena in the solution tested. For solutions with concentrations of 1% and 1.5%, the effect of temperature on relaxation was clearer and affected the shape of the autocorrelation function. Firstly, the time taken for the autocorrelation function to disappear increased. For a 1% solution, it was 1 s at 25–35 °C and 0.2 s at 40 °C. For a solution in the concentrated range (1.5%), the time interval was shifted ten times (1–10 s). Variations in the shape of the autocorrelation functions were also observed: at 25 °C and 30 °C, and at 35 °C and 40 °C, the functions were practically identical. Due to very long relaxation times, the values of the parameters of the KWW model (Equation (1)) could not be determined for higher concentrations. Different behavior to that observed for the 1.5% blackcurrant solution was seen in the case of the pectin solution obtained from redcurrants. Despite its significantly higher average molecular weight (1020 kDa), it was found that the autocorrelation function disappeared within the 0.01 to 0.1 s range in a manner similar to that observed for the 0.5% blackcurrant pectin solution. Relaxation times were determined from the autocorrelation functions shown in the figure using the KWW model, and diffusion coefficients were calculated based on these. The low values of *a*—describing the participation of fast modes in the hydrodynamic properties—showed the domination of slow relaxation phenomena. At 25 °C and 30 °C, the hydrodynamic radius values of the chains responsible for these phenomena exceeded 1000 nm.

**Figure 3:**
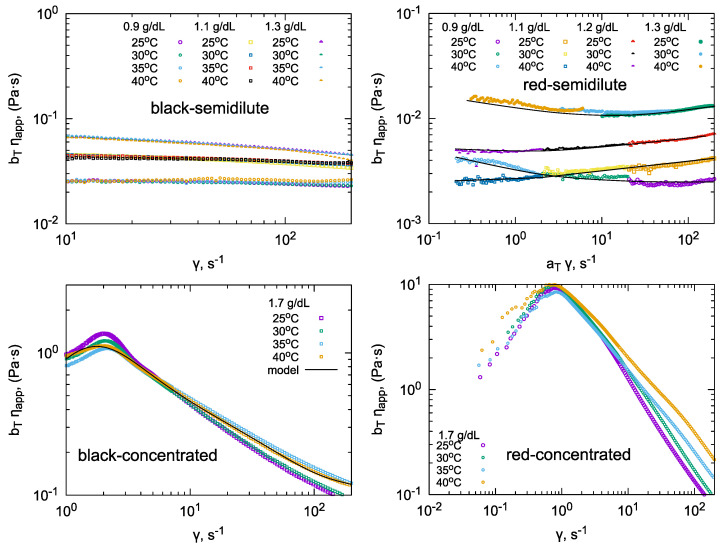
The apparent viscosity of pectin solutions at different concentrations as a function of shear rate and temperature. Data are shown for black and redcurrant pectin solutions in semidilute and concentrated regimes (top and bottom panels, respectively). Viscosity values were shifted using scaling coefficients: $a_{T}$ for shear rate and $b_{T}$ for apparent viscosity (see Table A1).

The rheological properties of the solutions within the flow range are presented in the graph (Figure 3). Blackcurrant pectin solutions exhibited Newtonian behavior, and the apparent viscosity–shear rate relationship could be scaled as a function of temperature. No scaling as a function of concentration was performed. In the concentrated solution regime, non-Newtonian behavior was observed, with a maximum on the flow curve. Consequently, the dependence of the apparent viscosity on the shear rate also exhibited a maximum, the position of which depended on the concentration and temperature of the solution.

Redcurrant pectin solutions exhibited complex, non-Newtonian rheological behavior in both the semidilute and concentrated regimes. The apparent viscosity–shear rate relationship for semi-dilute solutions could be scaled according to the temperature. The apparent viscosity of redcurrant pectin solutions in the semi-dilute range was an order of magnitude lower than that of blackcurrant pectin solutions. However, in the concentrated range (1.5–1.7%), the behavior was different: the values of $\eta_{app}$ were significantly higher.

## 3. Discussion

An increase in pectin concentration in the solution, regardless of its origin, and an increase in the temperature of the DLS measurement caused non-linear changes in the hydrodynamic behavior. Firstly, the proportion of short relaxation phenomena changed non-linearly with temperature; in both cases, this proportion was maximized at 30 °C. The effect of pectin concentration was much more complex and depended on the type of pectin. Comparisons can be made between the hydrodynamic properties of red and black pectin solutions within two concentration regimes. For blackcurrant pectin, an increase in concentration prolonged relaxation times; however, the only interpretable results were obtained at a concentration of 1.5% (see Table 2). Compared to redcurrant pectins, the pectins obtained from blackcurrant were characterized by a significantly lower average molecular weight ($M_{w}$ = 116 kDa) and a much higher degree of chain branching. Phenomenologically, the behavior of a 0.5% blackcurrant solution (semidilute regime) was similar to that of a 1.5% redcurrant pectin solution. Redcurrant pectins were characterized by a linear chain structure (R1 = 2.27) and an extremely high average molecular weight of 1020 kDa. Despite structural differences between these two pectins, the 0.5% autocorrelation function (blackcurrant semidilute) and the 1.5% autocorrelation function (redcurrant concentrated) were similar. However, differences appeared in the diffusion coefficient values: the $D_{f}$ values were higher for redcurrant pectin, while the $D_{s}$ values were of a similar order of magnitude. The differences in behavior became apparent with changes in temperature. In the 35–40 °C range, the hydrodynamic radius values ($R_{f}$ and $R_{s}$) for redcurrant pectins were lower than for blackcurrant pectins. Lowering the temperature to 25–30 °C caused an increase in $R_{h}$ values. For redcurrant pectins, $R_{s}$ values were greater than 1000 nm, while $R_{f}$ values reached their maximum at 30 °C. In the case of blackcurrant pectin (semidilute) solutions, the $R_{s}$ values were an order of magnitude higher than those of the concentrated 1.5% redcurrant solution.

Shear flow clearly affected the behavior of pectins in the semidilute region. Blackcurrant pectin exhibited semi-Newtonian behavior, with the temperature scaling of the apparent viscosity. This behavior suggests a phenomenologically simple mechanism that shapes rheological properties: friction. The blackcurrant solution had an apparent viscosity that was one order of magnitude higher than that of redcurrant pectin. Redcurrant pectin solutions exhibited non-Newtonian rheological properties that could not be analyzed using simple rheological models. However, the dependence of the apparent viscosity on the shear rate could be scaled using the coefficients $a_{t}$ and $b_{T}$ (see Table A1). In the concentrated regime, the rheological behavior of the two pectin solutions was similar, with both exhibiting a maximum in the apparent viscosity versus shear rate relationship. However, redcurrant pectin solutions exhibited significantly higher apparent viscosity values. In both cases, the apparent viscosity values were scaled using the $b_{T}$ coefficient.

An attempt was made to fit the Papanastasiou–De Kee model to the experimental data. Interpretable values of the model’s time constants were obtained for blackcurrant pectin solutions in the concentrated range (see Table A1). As the temperature increased, it was found that the characteristic times ($t_{i}$) also increased. This can be interpreted as an increase in the importance of friction-based mechanisms. An increase in pectin concentration at a constant temperature (25 °C) caused a decrease in $t_{i}$ values, except for $t_{3}$. This characteristic time corresponding to elastic contributions did not change significantly with increasing concentration. Its value is greater than one, which can be interpreted as indicating the presence of viscoelastic properties (the maximum on the apparent viscosity versus shear rate curve).

## 4. Materials and Methods

### 4.1. Pectins

Pectin was isolated from redcurrant *Rolan* and blackcurrant *Tissel* varieties purchased at a local market. Before use, berries were stored in freezing conditions (−20 °C).

### 4.2. Extraction

The currants were thawed at 4 °C for 24 h and used directly for further processing. Berries were homogenized in a blender until a uniform consistency was achieved. Due to their low pH, the obtained homogenates were mixed with distilled water in a ratio of 1:5 instead of citric acid. Pectin was extracted for 1 h and then filtered through filter paper. The clear filtrate was mixed with five times the volume of acetone. The precipitate, pectin was filtered off, rinsed with acetone, and dried in a laboratory oven at 40 °C. This method has been described by other authors as effective for isolating pectins from various raw materials [15,18]. Aqueous solutions of the samples were prepared at concentrations ranging from 0.9% to 1.7% (*w*/*w*). The weighed material was added to distilled water and then shaken for 3 h at 40 °C. The solutions were tested at temperatures ranging from 20 °C to 40 °C.

### 4.3. Molecular Weight Distribution

Molecular weight measurements were performed using the gel permeation chromatography (GPC) method. A system of two columns, Ultrahydrogel-2000 and Ultrahydrogel-500 (Waters, Milford, MA, USA), was used. As an eluent, a mixture of 0.1 M NaNO_3_ and 0.02% NaN_3_ solution in water with a flow rate of 0.6 mL·min^−1^ was applied. Calibration was performed using pullulan standards (Shodex, Resonac Corporation, Tokyo, Japan).

### 4.4. Dynamic Light Scattering (DLS)

Aqueous solutions of pectins were prepared by dissolving the pectin powder in deionized water; ionic strength or pH modifications were not used. The concentrations of the pectin samples were selected on the basis of previous experience to highlight the differences in the molecule’s properties. The samples were tested using dynamic light scattering at 25 °C with a Brookhaven DLS/SLS system consisting of a BI-160 goniometer with digital autocorrelator BI-9000AT (Brookhaven, New York, NY, USA). As the source of light, a solid-state laser (JDSU, CDPS532M-050, Huntingdon, UK) with an output power of 50 mW at λ = 532 nm was used. The light scattering angle chosen for the measurements was 90°. Three repetitions were performed for all samples. The time average intensity correlation function $\left[g^{2}\left(\tau \right)-1\right]$ was obtained with an acquisition time of 300 s for each run using Brookhaven Instruments Dynamic Light Scattering Software version 5.9. Mathematical modeling of hydrodynamic properties was carried out using the Kohlrausch–Williams–Watts (KWW) stretched exponential function [19]:

(1) $${\left[g^{2}\left(\tau \right)-1\right]}_{KWW}\approx {\left\{a\cdot exp\left(\frac{-\tau }{\tau_{f}}\right)+\left(1-a\right)\cdot exp{\left[-\left(\frac{\tau }{\tau_{s}}\right)\right]}^{\beta }\right\}}^{2}$$

where $g^{2}\left(\tau \right)-1$ is the intensity autocorrelation function; $\tau_{f}$, $\tau_{s}$ are the relaxation times of the fast (f) and slow (s) components, respectively; β is the exponent of the stretched exponential; τ is the delay time; and *a* and (1−a) represent the fractional contribution of the two processes. The parameters (Equation (1)) were estimated according to the Levenberg–Marquardt algorithm using the least squares method:

(2) $$\chi^{2}=\Sigma {\left\{\left[g^{2}\left(\tau \right)-1\right]-{\left[g^{2}\left(\tau \right)-1\right]}_{KWW}\right\}}^{2}⟶min$$

The diffusion coefficients for slow $D_{s}$ and fast $D_{f}$ components were calculated according to

(3) $$D_{k}=\frac{1}{\tau_{k}\cdot q^{2}},k=s,f$$

where $q=\frac{4\pi n}{\lambda }\cdot sin\left(\frac{\theta }{2}\right)$ is the value of the magnitude of the scattering wave vector.

### 4.5. Rheological Properties

The rheological characteristics of the aqueous solutions of blackcurrant and redcurrant pectins at various concentrations (0.9 g·dL to 1.7 g·dL) were determined using a rotational rheometer RS6000 (ThermoFisher, Karlsruhe, Germany) with a cone-plate geometry sensor ($d_{in}$ = 60 mm, 1°). Measurements were made at four temperatures: 25 °C, 30 °C, 35 °C and 40 °C. Three repetitions were performed for all samples. The rheological properties were described using the De Kee and Papanastasiou model [20,21]:

(4) $$\eta \left(\dot{\gamma }\right)=\tau_{0}{\dot{\gamma }}^{-1}+\eta_{1}\exp\left(-t_{1}\dot{\gamma }\right)+\eta_{2}\exp\left(-t_{2}\dot{\gamma }\right)-\eta_{3}\exp\left(-t_{3}\dot{\gamma }\right)$$

The estimated parameters were the yield stress $\tau_{0}$ (Pa), the exponents $t_{p}$ with time dimension, and coefficients $\eta_{p}$p=1,2 with the dimension of viscosity. In the case of estimating a larger number of pairs ($t_{p},\eta_{p}$), the results can be presented in the form of a discrete distribution of time constants $\eta_{p}\left(t_{p}\right)$. The coefficient values of $\eta_{p}$ describe the intensity of the time constants $t_{p}$. The parameters (Equation (4)) were estimated according to the Marquardt–Levenberg method, which was applied as a minimization algorithm using the least squares method. The target function is defined as follows:

(5) $$\chi^{2}=\Sigma {\left[\eta^{\sigma }-\hat{\eta }\right]}^{2}⟶min$$

where $\eta^{\sigma }$ are experimental values of apparent viscosity; $\hat{\eta }$ were calculated using Equation (4).

## 5. Conclusions

Analysis of the relaxation phenomena in pectin solutions indicates that their structure (linear or branched) affects their properties. Hydrodynamic studies revealed that relaxation phenomena occurred in a similar manner for redcurrant pectin in the concentrated region and for blackcurrant pectin in the semi-dilute region. Under shear flow conditions, differences in behavior were apparent even within the semidilute range: blackcurrant pectin solutions behaved like Newtonian fluids, whereas redcurrant pectin exhibited complex, non-Newtonian behavior. Redcurrant pectin solutions also exhibited lower apparent viscosity values at concentrations comparable to those of blackcurrant pectin.

The hydrodynamic and rheological properties of aqueous pectin solutions are determined by the conformation of the pectin chains in solution. The structure of polysaccharides (i.e., their average molecular weight or degree of chain branching) depends on their botanical origin. This study demonstrated that pectins obtained from two popular Polish currant varieties can yield polysaccharides with a similar degree of esterification and different chain structures. Characterizing pectins from different sources can reveal a variety of preparations that can be used in the food industry for different purposes.

## Figures and Tables

**Table 1:** Molecular properties of pectin obtained from red and blackcurrants and their first critical concentration ($c^{*}$, overlap concentration) according to [2,14,15].

Pectin	$M_{w}$	$M_{n}$	DE	R1	R2	R3	c*
	kg·mol^−1^	kg·mol^−1^	%				%
redcurrant	1020 ± 24	53 ± 2	57.1 ± 1.6	2.27	0.06	1.03	0.78
blackcurrant	116 ± 4	24 ± 1	67.4 ± 1.3	1.50	0.21	8.77	0.43

**Table 2:** The values of the KWW model in Equation (1) for semidilute solution of pectin from redcurrant (0.5%) and concentrated solution of pectin from blackcurrant (1.5%). The values of the $\chi^{2}$ statistic (Equation (2)—the goodness of model fit) did not exceed 10^−4^.

T, °C	a	β	$D_{f}$, ${\mathrm{cm}}^{2}/s$	$R_{f}$, nm	$D_{s}$, ${\mathrm{cm}}^{2}/s$	$R_{s}$, nm
redcurrant						
40	0.02 ± 0.00	0.7 ± 0.01	1.18 $\times \;10^{-11}$	30	7.95 $\times \;10^{-13}$	440
35	0.01 ± 0.00	0.7 ± 0.01	5.80 $\times \;10^{-12}$	54	5.20 $\times \;10^{-13}$	600
30	0.46 ± 0.01	0.9 ± 0.01	4.60 $\times \;10^{-13}$	61	7.47 $\times \;10^{-14}$	>1000
25	0.03 ± 0.00	0.7 ± 0.01	1.40 $\times \;10^{-11}$	16	1.13 $\times \;10^{-13}$	>1000
blackcurrant						
40	0.28 ± 0.01	0.7 ± 0.01	1.46 $\times \;10^{-12}$	150	5.32 $\times \;10^{-13}$	660
35	0.66 ± 0.02	0.8 ± 0.01	4.37 $\times \;10^{-13}$	490	2.14 $\times \;10^{-12}$	750
30	0.99 ± 0.05	0.9 ± 0.01	8.69 $\times \;10^{-13}$	250	7.29 $\times \;10^{-13}$	830
25	0.69 ± 0.02	0.9 ± 0.01	6.66 $\times \;10^{-13}$	330	5.48·$\times \;10^{-13}$	850

## Data Availability

The data are contained within the article.

## Appendix A

**Table A1:** Rheological properties of solutions at different concentrations of pectin from redcurrant and blackcurrant: the values of the De Kee and Papanastasiou model (Equation (4)): yield stress τ—Pa, time constants $t_{i}$—s, target function (Equation (5)) $\chi^{2}$, the values of scaling coefficients of $a_{T}$ for shear rate and $b_{T}$ for apparent viscosity. The table contains the estimated model parameter values, with an error margin of less than 20%. The goodness of fit of the presented models is described by the $\chi^{2}$ statistic.

Blackcurrant		25 °C	30 °C	35 °C	40 °C		25 °C	30 °C	35 °C	40 °C
0.9%		Newtonian fluid				$a_{T}$	1	1	1	1
						$b_{T}$	1	1.2	1.5	1.6
1.1%						$a_{T}$	1	1	1	1
						$b_{T}$	1	1.2	1.5	1.6
1.2%						$a_{T}$	1	1	1	1
						$b_{T}$	1	1.1	1.2	1.4
1.3%	$\tau_{0}$	0.16	1.2	1.9		$a_{T}$	1	1	1	1
	$t_{1}$	0.002	0.006	0.004						
	$t_{2}$	0.05	0.7	1						
	$t_{3}$	2.1	1.4	1.5		$b_{T}$	1	1	1	1
	$\chi^{2}$	$5\times 10^{-6}$	$2.8\times 10^{-4}$	$5.1\times 10^{-4}$						
1.5%	$\tau_{0}$	1.2	1.2	2.5	2.6	$a_{T}$	1	1	1.2	1.3
	$t_{1}$	0.002	0.002	0.001	0.003					
	$t_{2}$	0.033	0.05	0.04	0.1					
	$t_{3}$	1.6	1.6	1.8	2.6	$b_{T}$	1	1	0.6	0.45
	$\chi^{2}$	$1.9\times 10^{-4}$	$1.4\times 10^{-4}$	$2.5\times 10^{-4}$	$8.4\times 10^{-5}$					
1.7%	$\tau_{0}$	4.8				$a_{T}$	1	1	1	1
	$t_{1}$	0.018								
	$t_{2}$	0.003								
	$t_{3}$	2.1				$b_{T}$	0.6	0.7	1	1
	$\chi^{2}$	$2.1\times 10^{-4}$								
**Redcurrant**		**25 °C**	**33 °C**	**35 °C**	**40 °C**		**25 °C**	**33 °C**	**35 °C**	**40 °C**
0.9%						$a_{T}$	1	0.1		0.01
						$b_{T}$	1	1		1
1.1%		Shear-thickening				$a_{T}$	1	0.1		0.01
						$b_{T}$	1	1		1
1.2%		Shear-thickening				$a_{T}$	1	0.1		0.01
						$b_{T}$	1	1		1.1
1.3%		Shear-thinning				$a_{T}$	1	0.3		0.03
						$b_{T}$	1	1		1.6
1.5%	$\tau_{0}$	10		-	-	$a_{T}$	1	0.8	0.6	1
	$t_{1}$	0.045		-	-					
	$t_{2}$	0.41		-	-					
	$t_{3}$	2.55		-	-	$b_{T}$	1	2	6	1
	$\chi^{2}$	$1.8\times 10^{-3}$		-	-					
1.7%	$\tau_{0}$	-				$a_{T}$	1	1	1	1
	$t_{1}$	-								
	$t_{2}$	-								
	$t_{3}$	-				$b_{T}$	1	1.4	2.7	4.5
	$\chi^{2}$	-								
